# Comparison of the Different Treatment Strategies for Patients with Rectus Sheath Haematoma

**DOI:** 10.3390/medicina54030038

**Published:** 2018-05-30

**Authors:** Audrius Gradauskas, Linas Venclauskas, Matas Pažusis, Andrius Karpavičius, Almantas Maleckas

**Affiliations:** 1 Department of Nephro-Urology and Surgery, Clinic of Gastroenterology, Institute of Clinical Medicine, Faculty of Medicine, Vilnius University, LT-01513 Vilnius, Lithuania; andrius.karpavicius@gmail.com; 2 Department of Surgery, Lithuanian University of Health Sciences, LT-44307 Kaunas, Lithuania; linasvenclauskasg@yahoo.com (L.V.); ppazusis@gmail.com (M.P.); almantas_maleckas@yahoo.com (A.M.)

**Keywords:** rectus sheath haematoma, embolisation, ultrasound drainage of haematoma, conservative treatment

## Abstract

*Background and objective*: Rectus sheath haematoma (RSH) is an uncommon condition that may vary from contained haematoma to life-threatening bleeding. Timely diagnosis and treatment is crucial in this patient population. The aim of the current study was to investigate the results of the different RSH treatment strategies among patients admitted to a surgery department. *Materials and methods*: A retrospective analysis of 29 patients treated for RSH in surgery departments of two medical centres from 1 January 2007 to 30 September 2017 was conducted. The patient’s age, sex, ASA (American Society of Anesthesiologists; physical status classification system), use of anticoagulants, cause of haematoma, radiological data, vital signs, blood investigations, and type of treatment were extracted. The results were analysed according to the type of treatment. *Results*: The patients’ mean age was 67.6 ± 14.3 years, and the mean duration of in-hospital stay was 10.7 ± 6.7 days. All patients were on anticoagulant treatment, and 82.8% of them had spontaneous haematoma. Nine patients (31%) needed transfusion of packed red blood cells with an average of 2.6 units (range: 1–4). Five patients (17.2%) presented with symptoms and signs of hypovolemic shock, and four of them underwent embolisation. Embolisation was successful in all cases. Open surgery was performed in 6 patients, 8 patients underwent percutaneous drainage, and 10 patients were treated conservatively. Two patients (6.7%) died in our series. Both of these patients had type III RSH. Patients in the conservatively treated group had the shortest hospital stay. There were no readmissions due to repeated haematoma or infection. *Conclusions*: Embolisation of epigastric arteries is a useful tool to stop bleeding into RSH in patients with unstable haemodynamics. Conservative treatment is comparable to ultrasound (US) drainage of RSH but results in a shorter hospital stay. Type III RSH is associated with a higher death rate.

## 1. Introduction

Rectus sheath haematoma (RSH) is an uncommon condition that results from bleeding into the abdominal anterior rectus sheath. The main predisposing factors are blunt abdominal trauma and anticoagulant therapy [1]. Muscle tear and damage to epigastric arteries or its branches are the main sources of haemorrhage. RSH is frequently localised below the umbilicus where epigastric vessels are relatively fixed and prone to tearing [1]. Furthermore, between the umbilicus and pubis all the aponeuroses pass in front of the rectus abdominal muscle, thus creating a possibility for haematoma to spread between the rectus muscle and fascia transversalis into the prevesical space.

Generally, RSH is considered to be a self-limited condition. However, anatomical predispositions, particularly combined with anticoagulant treatment and epigastric artery injury, may result in massive haematomas and create a life-threatening situation [2]. Mortality from RSH in patients with anticoagulant therapy may reach up to 25% [3]. Timely diagnosis and treatment is crucial in this patient population, which consists mostly of elderly individuals with severe comorbidities.

The aim of the current study was to analyse the results of RSH according to computer tomography (CT) classification.

## 2. Material and Methods

**Figure 1:**
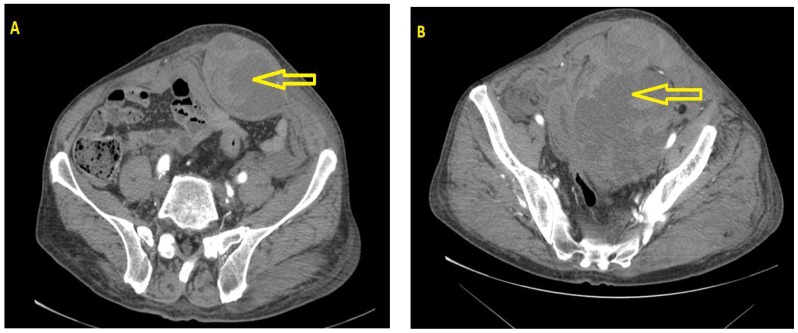
Type III rectus sheath haematoma (RSH) according to classification of Berna et al. [4]: (**A**) haematoma of rectal abdominal muscle; (**B)** haematoma proliferates to prevesical and retroperitoneal space. (The computer tomography (CT) scan images are from Department of Radiology, Lithuanian University of Health Sciences, Kaunas Clinics).

This was a retrospective analysis of patients ≥18 years old treated for RSH in the Departments of Surgery at Antakalnio Hospital (Vilnius) and the Lithuanian University of Health Sciences Hospital (Kaunas) from 1 January 2007 to 30 September 2017. The medical records were reviewed for the diagnosis of abdominal wall haematoma or abdominal wall haemorrhage. RSH was diagnosed if the main location of the haematoma was in the rectus sheath with or without spread to the prevesical space or peritoneum and was confirmed by radiological investigations (abdominal ultrasound (US) or CT). Twenty-nine patients met the diagnostic criteria and were included in this study. The patients’ age, sex, ASA (American Society of Anesthesiologists; physical status classification system) class, use of anticoagulants, cause of haematoma, radiological data, vital signs, blood investigations, and type of treatment (angiography with embolisation, open surgery, percutaneous procedures, and conservative treatment) were extracted. Haematomas were graded according to the classification of Berna et al. [4]: type I haematoma (intramuscular with minimal to no haemodynamic compromise); type II haematoma (intramuscular, but with blood between the rectus abdominal and the transversalis fascia); type III haematoma (blood extending to the peritoneum and the prevesical space—Figure 1). The results were analysed according to the type of treatment.

SPSS 21 software (IBM Corporation, Armonk, NY, USA) was used for statistical analysis. The mean (standard deviation) was used to present continuous variables, and categorical variables were expressed as the number (percentage) of patients affected. Normality of the data was tested by the Shapiro–Wilk test. Continuous variables between the groups were compared using one-way ANOVA with the Bonferonni post hoc test. The chi-square test was used to compare categorical variables. A significant difference was considered when the *p*-value was below 0.05.

To perform this retrospective study, it was approved by the Ethical Committee (8 May 2018; No. 158200-18/5-1035-532).

## 3. Results

Twenty-nine patients were included in the study. The mean age of the patients was 67.6 ± 14.3 years. Six (20.7%) patients were males, and 23 (79.3%) patients were females. The mean duration of the patients’ in-hospital stay was 10.7 ± 6.7 days. All patients were on anticoagulant treatment: 79.4% on warfarin, 3.4% on bemiparin sodium, and 17.2% on acetylsalicylic acid. Twenty patients (68.9%) used anticoagulants because of dysrythmias or previous cardiac surgery. Nine patients (31.1%) had anticoagulant medications for previous deep vein thrombosis, pulmonary embolism, or hipercoagulopathy. The main reason for RSH was spontaneous haematoma in 82.8% of cases. Four patients (13.8%) had mild trauma of the abdomen, and one (3.4%) developed RSH after injection of anticoagulants.

In 26 and 3 cases, respectively, abdominal US and abdominal CT were performed as an initial radiological investigation. In 17 cases, in addition to US, abdominal CT was performed. After CT, RSH was graded according to the classification of Berna et al. [4]: type I haematoma was present in four patients; type II haematoma was present in nine patients; type III haematoma was present in seven patients.

All patients were treated with intravenous fluid resuscitation. The international normalized ratio (INR) was on average 2.4 (range: 1.0–8.2) among the 23 patients who were using warfarin. Five patients (21.7%) had an INR of >3.0. Reversal of anticoagulation was performed in 11 patients (47.8%) when the INR exceeded 2, either with fresh frozen plasma and vitamin K in 5 cases or with 4-factor prothrombin complex concentrates (Octaplex) in 6 patients. Nine patients (31%) needed transfusion of packed red blood cells with an average of 2.6 units (range: 1–4). Five patients (17.2%) presented with symptoms and signs of hypovolemic shock, and four of them underwent embolisation. Overall, angiography and epigastric artery embolisation with coils was performed in five cases. Open incision with evacuation of haematoma was performed in three patients, and evacuation through laparotomy was performed in another three cases. Eight patients underwent percutaneous drainage, and 10 patients were treated conservatively.

There was no difference between the groups regarding age, sex, ASA class, anticoagulant use, or cause of haematoma (Table 1). Patients in the embolisation group had significantly lower diastolic blood pressures and increases in urea as a sign of active haemorrhage (Table 2). The greatest average size of haematoma was also seen in the embolisation group, and the patients with type III haematoma were prevalent in the embolisation and surgery groups (Table 3). Patients in the conservatively treated group had the shortest hospital stay (Table 4). Anticoagulation therapy with low-molecular-weight heparins (LMWHs) was reintroduced after stabilising patients’ haemodynamic conditions, and the dose was judged according to the risk of tromboembolism. Oral anticoagulants were administered 7–10 days after LMWH introduction. No patient needed re-hospitalisation due to additional bleeding or infection of RSH.

Two patients (6.7%) died in our series. One patient had contrast extravasation on their CT scan, but angiography and embolisation were not performed. The patient was treated conservatively and died from haemorrhagic shock. The other patient had injury to the epigastric artery after injection of LMWH and underwent embolisation of the epigastric artery and later percutaneous drainage of abdominal wall haematoma. The bleeding stopped, but the patient died from progressive cardiovascular and renal failure. Both patients had type III haematoma. Thus, the mortality rate among the patients with type III haematoma reached 28.6%.

## 4. Discussion

The clinical presentation and extent of RSH is diverse, and patients may need different management approaches. When acute haemorrhage is present with signs of haemodynamic instability, patients need angiography and embolisation to stop bleeding and to stabilise the condition. The patients in our study who underwent angiography and embolisation had significantly lower diastolic blood pressure and increased urea as signs of severe hypovolemia due to bleeding. In all cases, embolisation was successful. Other studies also support the view that angiography and embolisation of epigastric arteries is highly effective in controlling bleeding into RSH [5,6,7]. The overall mortality rate in our study was 6.8%, similar to the data from a recent study that also used arteriography for patients with active bleeding [5].

Abdominal US is a useful diagnostic tool, as RSH may account for up to 2% of cases with unexplained abdominal pain [3,8]. However, the sensitivity and specificity of US are lower in comparison to those of abdominal CT. Moreover, CT may allow for a more precise classification of haematoma [4] and currently is considered to be the “gold standard” for diagnosis and evaluation of RSH [9]. In our study, most of the patients had abdominal US first followed by CT scan. US investigation in patients with abdominal pain in our institutions is a routine practice and is supported by some published guidelines [10]. If a haematoma is contained to the rectus muscle and does not show signs of progression, CT scan can be avoided, as it was in half of our conservatively treated patients. However, when a patient’s haemodynamics is unstable or either surgery or percutaneous drainage is planned, CT is a valuable investigation. The sensitivity of CT angiography for depicting bleeding from the arteries in soft tissues is as high as 87% [6].

Similarly to other series, open surgery in our patients was performed mainly for type III haematoma [11], and half of the patients had laparotomy on the first day of admission, because rupture of the RSH and intraperitoneal bleeding were suspected. All patients who underwent open surgery were haemodinamically stable, and there were no signs of active bleeding during surgery in any case. Currently, open surgery is not considered as a first-line treatment, because it eliminates the tamponade effect, and the identification of a bleeding source may be problematic [5,6]. However, patients with suspected intraperitoneal bleeding should be regarded as candidates for acute surgery. Contained type III haematomas in haemodinamically stable patients do not require immediate intervention. However, there are no prognostic factors that could predict haemodynamic instability [11]. Consequently, these patients should be monitored closely by contrast-enhanced CT examinations and should be treated aggressively [11]. As shown by our study, mortality among the patients with type III haematoma reached up to 28.6%. Intra-abdominal pressure should be routinely measured in such patients, because abdominal compartment syndrome with secondary renal failure has been reported. The timely evacuation of haematomas may restore renal function and improve survival among patients with RSH and abdominal compartment syndrome [12].

The other group is patients with type I and II haematoma. Whether these haematomas should be treated conservatively or by percutaneous drainage is unclear. US drainage and conservative treatment groups in our study were similar in terms of all parameters, including haematoma size. The in-hospital stay among the patients with US drainage was twice as long as for those in the conservatively treated group. There were no serious complications associated with haematoma, and nobody was readmitted to the hospital in either group. Thus it seems reasonable that US drainage should be avoided when possible, as it has a potentially higher risk for infection [5]. Usually, the complete resolution of a haematoma occurs within 1–2 months [13].

The reintroduction of anticoagulants in patients with RSH is an important topic. In our study, we tried to administer LMWH as early as possible. We did not observe repeated haematoma or thromboembolic complications. However, some studies confirm the possibility of repeated episodes of haematoma after the reintroduction of anticoagulant treatment [11]. The decision must be taken on the basis of common sense and by weighting the risk of bleeding against the risk of thromboembolic complications.

RSH in our cohort was more common in women, similarly to the results from other published studies [9,11,14]. This may be attributed to the difference in muscle mass between genders [15]. Men have a larger muscle mass, which better protects muscle itself and associated vessels in the case of trauma. However, there is no good explanation for why females are more prone to the development of spontaneous haematoma.

RSH may occur at any age in adult life but predominantly affects the elderly, as the number of risk factors for RSH increases with age. In the majority of our patients, there was no evident relation of haematoma to trauma, and these cases were named spontaneous RSH. These patients had either warfarin or antiplatelet medications, which are predisposing risk factors for RSH. Coughing paroxysm, vomiting, and strenuous urination or defecation increase intra-abdominal pressure and, in combination with anticoagulants, facilitate the development of RSH [5]. Because of the retrospective nature of our study, we were unable to explore these triggering risk factors. Mild trauma as a cause of RSH was considered if there was a history of physical exertion; a fall; or haematoma appeared after reaching, twisting or lifting; it was present in 13.8% of our patients. One patient had injury of the epigastric artery from a needle while injecting LMWH. A similar case was earlier described in the literature [16]. Despite the fact that such injuries are extremely rare, one must be aware of the possibility to damage the epigastric artery during the injection of LMWH.

The current study has some drawbacks. First, the data are presented for a selected population of patients admitted to the surgery department. Second, the study is retrospective in nature, with a small number of patients in different treatment groups, which makes statistical comparison of treatment strategies more difficult. Finally, not all patients had CT to classify RSH. However, despite the small sample size, our study is among the first to compare the results of different treatment options according to the type of RSH. More studies are needed to define the role of surgical or US drainage in haemodynamically stable patients with contained haematomas.

## 5. Conclusions

The embolisation of epigastric arteries is a useful tool to stop bleeding into RSH in patients with unstable haemodynamics. Conservative treatment is comparable to US drainage of RSH, but results in a shorter hospital stay. Type III RSH is associated with a higher death rate.

## Figures and Tables

**Table 1:** Patients’ characteristics, anticoagulants used, and cause of haematoma.

Parameters	Embolisation (*n* = 5)	Open Surgery (*n* = 6)	US Drainage (*n* = 8)	Conservative Treatment (*n* = 10)	*p*-Value
Age (years)	72.8 (SD: 9.1)	73.7 (SD: 10.8)	67.7 (SD: 14.4)	61.3 (SD: 17.0)	0.310
Sex: M/F (n)	1/4	3/3	1/7	1/9	0.244
ASA (n)					0.214
2	1	0	4	5	
3	4	4	4	5	
4	0	1	0	0	
5	0	1	0	0	
Anticoagulants					0.561
Warfarin (n)	4	6	7	6	
LMWH (n)	0	0	0	1	
Antiplatelet (n)	1	0	1	3	
Cause of haematoma					0.513
Spontaneous (n)	5	4	7	8	
Mild trauma (n)	0	1	1	2	
Injection (n)	0	1	0	0	

US drainage: ultrasonography drainage; SD: standard deviation.

**Table 2:** Pulse, blood pressure, and laboratory investigations on admission.

Parameters	Embolisation (*n* = 5)	Open Surgery (*n* = 6)	US Drainage (*n* = 8)	Conservative Treatment (*n* = 10)	*p*-Value
HR (beats/min)	101.6 (SD: 8.2)	85.8 (SD: 8.5)	88.0 (SD: 10.9)	90.1 (SD: 19.6)	0.282
SBP (mmHg)	107.0 (SD: 28.5)	133.8 (SD: 18.8)	132.0 (SD: 26.0)	135.9 (SD: 31.3)	0.268
DBP (mmHg)	49.6 (SD: 14.3) *^,§^	78.3 (SD: 8.9) *	71.9 (SD: 16.2)	75.4 (SD: 15.3) ^§^	0.011
WBC (×10⁹/L)	12.3 (SD: 2.5)	8.5 (SD: 3.6)	9.0 (SD: 3.2)	10.4 (SD: 2.5)	0.157
Hb (g/L)	100.0 (SD: 22.3)	108 (SD: 18.3)	103.5 (SD: 21.4)	112.7 (SD: 26.2)	0.733
Ht (%)	23.0 (SD: 14.1)	33.2 (SD: 5.3)	31.8 (SD: 6.0)	26.7 (SD: 15.5)	0.409
Urea (mmol/L)	13.8 (SD: 6.7) ^¶,^^◆^	7.0 (SD: 4.7)	5.9 (SD: 3.1) ^¶^	6.1 (SD: 1.8) ^◆^	0.007
Creatinine (mmol/L)	317.8 (SD: 206.8) °”	189.7 (SD: 236.1)	104.4 (SD: 39.8) °	104.8 (SD: 51.1) ”	0.041
INR	1.9 (SD: 1.0)	3.0 (SD: 2.7)	2.8 (SD: 1.2)	1.8 (SD: 0.4)	0.489
DATL (sec.)	33.6 (SD: 9.0)	69.7 (SD: 61.4)	38.2 (SD: 5.4)	57.9 (SD: 53.3)	0.435

US drainage: ultrasonography drainage; SD: standard deviation; HR—heart rate; SBP—systolic blood pressure; DBP—diastolic blood pressure; * *p* = 0.017; ^§^ *p* = 0.019; ^¶^ *p* = 0.010; ^◆^ *p* = 0.009; °” *p* = 0.041.

**Table 3:** Computer tomography (CT) findings on admission.

Parameters	Embolisation (*n* = 5)	Open Surgery (*n* = 4)	US Drainage (*n* = 6)	Conservative Treatment (*n* = 5)	*p*-Value
Haematoma size (mL)—CT	940.0 (SD: 371.5)	612.5 (SD: 225.0)	650.0 (SD: 209.8)	720.0 (SD: 525.1)	0.498
Haematoma classification (*n*)					0.007
Type I	0	0	1	3	
Type II	3	0	5	1	
Type III	2	4	0	1	

US drainage: ultrasonography drainage; SD: standard deviation.

**Table 4:** Treatment outcomes between the groups.

Parameters	Embolisation (*n* = 5)	Open Surgery (*n* = 6)	US Drainage (*n* = 8)	Conservative Treatment (*n* = 10)	*p*-Value
Packed red blood cells (units)	1.8 (SD: 1.5)	1.0 (SD: 1.7)	0.25 (SD: 0.7)	0.6 (SD: 1.3)	0.221
Intervention (days after admission)	0	1.7 (SD: 1.2)	5.2 (SD: 4.4)	—	—
In-hospital stay (days)	9.4 (SD: 4.0)	16.3 (SD: 10.6) *	12.4 (SD: 5.0)	6.7 (SD: 3.2) *	0.028
Mortality	1	0	0	1	0.471

US drainage: ultrasonography drainage; SD: standard deviation; * *p* = 0.027.
